# Neurological safety of spinal surgery for nucleus pulposus removal under spinal endoscopic imaging guided by inter laminar spine

**DOI:** 10.12669/pjms.37.6-WIT.4880

**Published:** 2021

**Authors:** Bin Chen, Zengfeng Du

**Affiliations:** 1 Bin Chen, Master of Medicine, Associate Chief Physician, Department of Orthopaedics, Yulin No.2 Hospital, Yulin, 718000, Shaanxi, China; 2 Zengfeng Du, Master of Medicine, Associate Chief Physician, Department of Orthopaedics, The First Hospital of Yulin, Yulin, 718000, Shaanxi, China

**Keywords:** Spinal Surgery, Spinal Endoscopic Imaging, Inter Laminar Spine, Nucleus Pulposus Removal

## Abstract

**Objective:**

To explore the technical points, approach selection and short-term clinical efficacy of PELD through the intervertebral foramina or interlaminar approach in the treatment of highly shifted LDH.

**Methods:**

From September 2018 to June 2020, 19 patients with highly shifted LDH were treated with PELD in The First Hospital of Yulin. It included, 10 males and 9 females; aged 34 to 69 years, with an average of 48 years. Thirteen cases were shifted to the caudal side, and six cases were shifted to the head side. The responsible segments included L3/41 cases, L4/511 cases, and L5/S17 cases. All patients had symptoms of low back and leg pain. The Sowerby dysfunction index (ODI) was 63.5%±10.7% before surgery. The visual analogue scale of pain (VAS) was low back pain (5.2±2.1) and leg pain (7.1±2.4). 14 cases used transforaminal approach, and 5 cases used translaminar approach.

**Results:**

All cases completed the operation successfully, the operation time was 60~110min, with an average of 70 minutes. The follow-up time ranged from 6 to 42 months, with an average of 20.8 months. At the last follow-up, ODI was 10.8%±6.8%, VAS back pain score (2.1±1.1) and leg pain score (1.8±0.9). Compared with preoperative, ODI and VAS scores were significantly decreased (P<0.05). The results of Mac Nab method were 14 excellent, four good, and one fair. During the follow-up period, one patient’s leg pain symptoms recurred seven days after operation. No further hernia was found under intervertebral foramen. The symptoms disappeared after two weeks of symptomatic treatment such as swelling and analgesia, and he was discharged. No perioperative complications such as infection and nerve root injury occurred.

**Conclusion:**

When PELD is used to treat high-displacement LDH, the choice of transforaminal approach or interlaminar approach needs to be personalized according to the LDH segment and the direction of displacement.

## INTRODUCTION

The clinical incidence of disc herniation is high.^1^ Endoscopic extraction of spinal nucleus pulposus is a more widely used minimally invasive operation modality in clinical application. It has the advantages of little intraoperative blood loss, small trauma area, quick postoperative recovery of patients, and obvious therapeutic effect.^2^ With the development of minimally invasive techniques, percutaneous endoscopic lumbar nucleus pulposus excision (PELD) has become more and more mature. Transforaminal and translaminar approach PELD has been used in the treatment of various types of lumbar disc herniation (LDH) with good clinical efficacy.^3^,^4^ Studies indicated that the therapeutic effect of endoscopic spinal nucleus pulposus extraction was similar to that of traditional open discectomy.^5^-^7^

Some researchers have gradually applied MELD to LDH with high displacement to ensure surgical efficacy and further reduce surgical trauma.^8^-^10^ The biggest risk in removing the lumbar disc is damage to the nerve roots. A clear understanding of the anatomical relationship between the spinal canal nerve and its adjacent structures is the key to reduce intraoperative nerve injury.^11^-^13^ At present, there are relatively few studies to evaluate the neuro safety of translaminar intervertebral disc resection for disc herniation, and very few neuroanatomical data are available.

Since September 2012, our department has applied PELD through the intervertebral foramen or interlaminar approach to highly displaced LDH and achieved good results. This study intends to explore the feasibility and safety of PELD in the treatment of highly shifted LDH, analyze the short-term efficacy after surgery; discuss the selection strategy of the surgical approach, and analyze the technical points, advantages and disadvantages of each approach.

## METHODS

From September 2018 to June 2019, 19 patients with highly shifted LDH were treated with PELD in The First Hospital of Yulin after IRB approval (dated March 14, 2021), including 10 males and 9 females; aged 34 to 69 years, with an average of 48 years.

### Inclusion criteria

A. Patients diagnosed with high-displacement LDH according to the definition of Choid, with low back or hip pain, combined with sciatica in one or both lower limbs; B. Conservative treatment for more than 3 months with poor results or repeated symptoms Onset; C. Preoperative Sowerby dysfunction index (ODI)> 30%; D. Preoperative visual analogue scale (VAS) for low back pain and leg pain> three points; E. CT, MRI, lumbar spine hyperextension flexion X The radiograph showed the imaging findings without lumbar instability.

### Exclusion criteria

A. disc herniation, protrusion, or mild prolapse; B. disc herniation with more than one responsibility segment; C. imaging suggestion of lumbar spine instability.

The segments of the responsible eight cases on the tail side), L5/S17 cases (two cases on the head side, five cases on the tail side). Preoperative X-ray films showed no obvious lumbar spine instability; CT all showed compression and displacement of the nerve roots of the responsible segment, and compression and deformation of the Dural sac; MRI showed grades II to III of the responsible segment intervertebral disc.

### Surgical methods

It is theoretically applicable to all types of nucleus pulposus prolapse and displacement in all segments, but the advantage is more obvious in L5/S1. The patient was in a prone position with the abdomen hanging. Continuous epidural anesthesia was used. X-ray vertical fluoroscopy locates the L5/S1 interlaminar space, and determines the puncture point according to the displacement of the nucleus pulposus toward the head or tail. Routine disinfection and towel laying. Cut the skin about seven mm at about one cm beside the spinous process, insert the dilation guide, and then insert the working tube. C-arm X-ray machine perspective confirms that the working pipe is in the correct position. Connect the endoscopic, endoscopic bipolar radiofrequency and intraoperative physiological saline lavage interface. Under the microscope, the nucleus pulposus forceps remove the soft tissue at the interlaminar space and expose the ligamentum flavum at the interlaminar space. Under the microscope, blue forceps bite the yellow ligament, revealing the dura mater and nerve root. Bipolar radiofrequency blunt separation of the dura mater and the posterior longitudinal ligament, the tip of the working pipe is rotated between the dura mater and the nerve root or on the shoulder, and the nucleus pulposus is removed under the microscope Dearmel area of nerve root. After bipolar radiofrequency hemostasis, properly withdraw from the working pipeline, check for the presence of nucleus pulposus tissue, and re-bipolar radiofrequency hemostasis. Withdraw from the working pipeline, sew one needle through the incision, and cover it with a sterile small dressing.

### Automatic correction algorithm for endoscopic images

The radial deformation of the endoscopic image is severe, while the tangential deformation is negligible. Its deformation model can be described as:


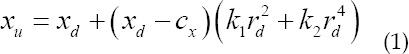



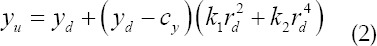


**Fig.1:**
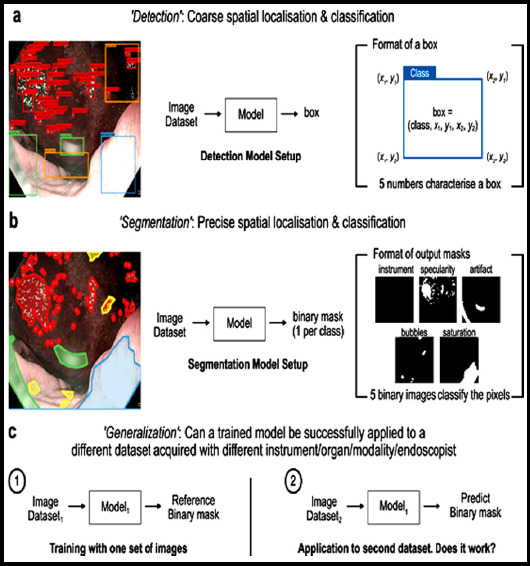
Process of automatic correction algorithm for endoscopic images.


Among them, *(x_d_,y_d_)^T^* and *(x_u_,y_u_)^T^* are distortion coordinates and correction coordinates; *(c_x_,c_y_*,*k*_1_,*k*_2_,*S_x_*)*^T^* is the deformation parameter; *r_d_* is the deformation radius. The corner points in *L_ij_* are located on the same straight line in the real physical world, but due to the radial deformation of the image, they are in the image the above are not collinear. In order to evaluate the degree of their collinearity, a straight line *ax+by+c*=0 is fitted to *L_ij_* solve. Fig.1 shows the flow of the algorithm for automatic correction of endoscopic images.

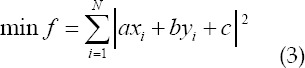


Take the residual of Eq. (9) as the straightness of set *L_ij_*. If *L_ij_* is strictly collinear, the greater the deformation of ^min *f* = 0 *L_ij_*^ the greater the ^min *f*^. The actual calculation does not need to solve for *(a,b,c)^T^*. Note that f is a quadratic about *(a,b,c)^T^* function:


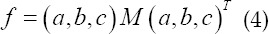


Is the number of corner points in *L_ij_*. Since *(a,b,c)^T^* has scale invariance (*Uptoscale*), the limit is:


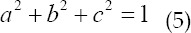


The minimum value of Eq. (4) under the restriction of Eq. (5) is the smallest eigenvalue of the three non-negative eigenvalues of the semi-positive definite symmetric matrix M, denoted as *λ_3_(M)*, and its corresponding unit eigenvector is denoted as *u_3_(M)*, denoted by:


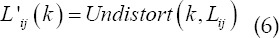


Is the set of points corrected for *L_ij_* using equations (6) and (7), where:


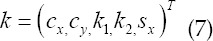


For all elements in L, construct the following objective function:


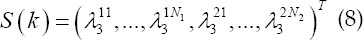


In this paper, the Levenberg-Marquardt algorithm is used for nonlinear optimization. In order to effectively optimize the iteration, the Jacobian matrix of S(k) needs to be given.

### Postoperative management and follow-up

After bed rest for two hours after operation, wear a soft waist to get out of bed, avoid bending and weight-bearing, and avoid squat toilet for one month and instruct patients to perform functional exercises of the lower back muscles and straight leg raising exercises on the bed. At 1, 3, 6, and 12 months postoperatively, the clinic was reexamined to evaluate the VAS back pain and leg pain scores and the ODI score. The efficacy was evaluated according to MacNab clinical evaluation criteria.

### Statistical analysis

Statistical analysis was performed using SPSS13.0 statistical software (SPSS, USA). The measurement data were expressed as mean ± standard deviation. The comparison of VAS back pain score, VAS leg pain score, and ODI at pre-operation, one month after operation, and last follow-up was performed by analysis of variance of repeated measurement data.

## RESULTS

### General situation of perioperative period

The operation time of this group was 60~110min, with an average of 70min. All patients with L3/4 and L4/5 prolapse and displacement and two patients with L5/S1 prolapse and downward displacement adopted transforaminal approach, two patients with L5/S1 prolapse and 3 patients with L5/S1 prolapse Patients with prolapse and displacement and high iliac crest use laminar space approach. One patient’s leg pain symptom reappeared seven days after surgery. In the remaining 18 cases, the nerve root and the Dural sac were fully decompressed during the operation, and no perioperative complications such as infection and nerve root injury occurred.

### Note

The patient underwent endoscopic L5/S1 nucleus pulpectomy through the interlaminar approach, and the postoperative MacNab method was effective A. The preoperative MRI sagittal T2WI showed that the nucleus pulposus prolapse of L5/S1 disc moved upward and exceeded L5. /S1 intervertebral space height, nucleus pulposus signal decreased; B. preoperative MRI cross-sectional T2WI showed prolapsed nucleus pulposus tissue compression of the right nerve root and Dural sac; C. preoperative CT showed LDH, no obvious calcification; D. Intraoperative picture, the working channel points to the caudal side; E. Intraoperative perspective prompts that the working channel tip is inclined to the head side, the working channel is located above the intervertebral space, and the range of intraoperative decompression has reached the upper vertebral body pedicle Margin; F. large amount of prolapsed nucleus pulposus tissue removed during operation; G, H. Postoperative MRI review showed that nerve root and Dural sac obtained good decompression; I. J. six months postoperative review showed fiber The annulus was well repaired and there was no recurrence of disc herniation.

### Evaluation of efficacy

Nineteen patients were followed up for 6 to 42 months, with an average of 20.8 months. Postoperative VAS back pain score, VAS leg pain score, and ODI were all lower than before operation, and the difference was statistically significant. There was no statistically significant difference between the last follow-up and 1 month after surgery (Table-I). The results of MacNab method were 14 excellent, four good, and one fair.

**Table-I:** VAS and ODI score results before, after and last follow-up.

*Observation index*	*Preoperative*	*Immediately after surgery*	*6 months after operation*
Low back pain VAS score	5.2±2.1	3.5±1.4	2.3±1.1
Leg pain VAS score	7.1±2.4	2.9±0.9	1.9±0.9
OD (I%)	63.5±10.7	20.6±6.8	13.5±6.9

## DISCUSSION

To improve the clinical effect of endoscopic resection of the nucleus pulposus, physicians need to choose a reasonable approach. In this research, the excellent and good effects of clinical treatment, VAS, and ODI scores were analyzed. VAS is mainly used to score the state of pain, with 0 indicating no pain and 10 representing the most intense unbearable pain.^14^,^15^ Lumbar Oswestry disability index (ODI) is used to score lumbar function and pain, and the higher the OSI score, the more severe the lumbar dysfunction of patients.^16^-^19^ The results showed that VAS and ODI scores were significantly lower than before treatment, which indicated that the pain and lumbar function of patients with disc herniation were significantly improved after the use of spinal endoscopic nucleus pulposus extraction, which was basically consistent with the research results of Zhang et al.^20^ Follow-up results showed that there were no perioperative complications such as infection and nerve root injury. In short, the endoscopic resection of the nucleus pulposus for the treatment of disc herniation has excellent neurological safety and is worthy of clinical adoption.

## CONCLUSIONS

The follow-up results of the patients confirmed that PELD can achieve satisfactory clinical efficacy in the treatment of highly shifted LDH, with less surgical trauma and fewer complications. However, the study has small number of cases. As the number of cases increases, more types of surgical complications may occur. This operation requires a high degree of endoscopic operation technique, and it needs to be carefully selected before obtaining a wealth of clinical experience.

### Author`s Contribution:

**BC** conceived the study, literature review, data analysis, drafting the manuscript.

**ZD** takes the responsibility and is accountable for all aspects of the work in ensuring that questions related to the accuracy or integrity of any part of the work are appropriately investigated and resolved.
